# COVID-19 Myopericarditis With Pericardial Effusion Complicated With Cardiac Tamponade and Rhabdomyolysis

**DOI:** 10.7759/cureus.27291

**Published:** 2022-07-26

**Authors:** Wael Abdelmottaleb, James Thomas Salmon, Bryan S Quintanilla Rodriguez, Ingrid Portillo, Savi Mushiyev

**Affiliations:** 1 Internal Medicine, Metropolitan Hospital Center - New York Medical College, New York City, USA; 2 Cardiology, Metropolitan Hospital Center - New York Medical College, New York City, USA

**Keywords:** acute pericardial effusion, rhabdomyolysis, myo-pericarditis, tamponade, covid 19

## Abstract

COVID-19 infection is a complex multi-organ disease, including the cardiovascular system, which may present with myocarditis. A 42-year-old female presented to our ED with generalized weakness, myalgia, and epigastric pain. Laboratory workup showed a positive SARS-CoV-2 polymerase chain reaction (PCR). An ECG showed sinus tachycardia with low voltage. A bedside echocardiogram showed a pericardial effusion with cardiac tamponade. An emergent pericardiocentesis was performed with immediate hemodynamic improvement. The patient was admitted to the coronary care unit (CCU), and colchicine and ibuprofen were started for pericarditis. Pericardial fluid bacterial and fungal cultures were negative, and serum antinuclear antibodies were also negative. On day 5 of hospitalization, creatine kinase (CK) level was high compared to on presentation. COVID-induced rhabdomyolysis was suspected and was dramatically improved with IV fluids. The patient was discharged on day 7 of admission. Our case shows that COVID-19 can present with an uncommon presentation like cardiac tamponade. Further studies are warranted to better understand the pathogenesis and management of COVID-19 myopericarditis.

## Introduction

COVID-19 infection is a complex multi-organ disease, including the cardiovascular system, which may present as myocardial infarction, myocardial injury, arrhythmias, heart failure, pericarditis, thromboembolic manifestations, and myocarditis. A large proportion of COVID-19-infected patients show raised cardiac biomarkers such as troponins and brain natriuretic peptide (BNP) [1].

Myocarditis is an uncommon presentation. Its occurrence is estimated between 2.4 and 4.1 out of 1000 patients hospitalized for COVID-19 infection. The majority of acute myocarditis occurs in the absence of pneumonia and is often complicated by hemodynamic instability [2]. Approximately 10% of cases of COVID-19 myocarditis have pericardial effusion. The pericardial fluid is usually free of virus, therefore likely representing an inflammatory rather than infectious process. Furthermore, the degree of the pericardial inflammatory response and the volume of pericardial effusion does not necessarily correlate with the degree of myocardial involvement. Therefore, a large pericardial effusion with tamponade can be seen in the absence of severe myocarditis [3]. Also, rhabdomyolysis associated with COVID-19 has been reported in a few studies [4].

## Case presentation

A 42-year-old female with no past medical history presented to the ED with three days of generalized weakness, myalgia, and epigastric pain. The patient denied any surgical or significant family history and was currently not on any medications.

**Figure 1:**
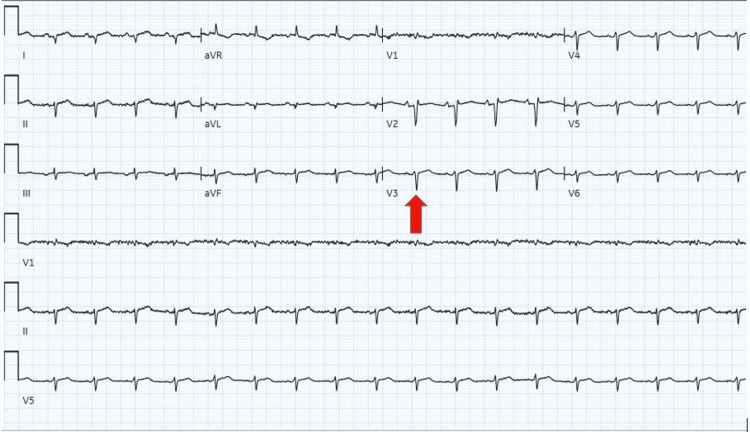
EKG on presentation: sinus tachycardia, poor R-wave progression, and low voltage (red arrow).

The vital signs showed hypotension and tachycardia, and the physical exam showed cold extremities, jugular venous distention, and distant heart sounds with friction rub. Laboratory workup showed a positive SARS-CoV-2 polymerase chain reaction (PCR), leukocytosis, erythrocytosis with hemoglobin 18.3, hyponatremia 129 mEq/L (normal 135-145), elevated troponin T 0.032 (normal <0.010), and mildly elevated liver enzymes. EKG showed sinus tachycardia with poor R-wave progression and low voltages (Figure 1). A bedside echocardiogram showed a pericardial effusion with cardiac tamponade. The patient had not received a COVID-19 vaccination.

**Figure 2:**
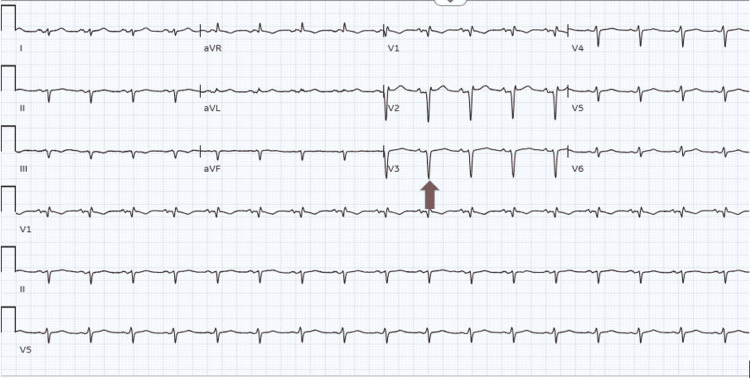
EKG after pericardiocentesis: sinus rhythm, poor R-wave progression, and improvement of low voltage (red arrow).

**Figure 3:**
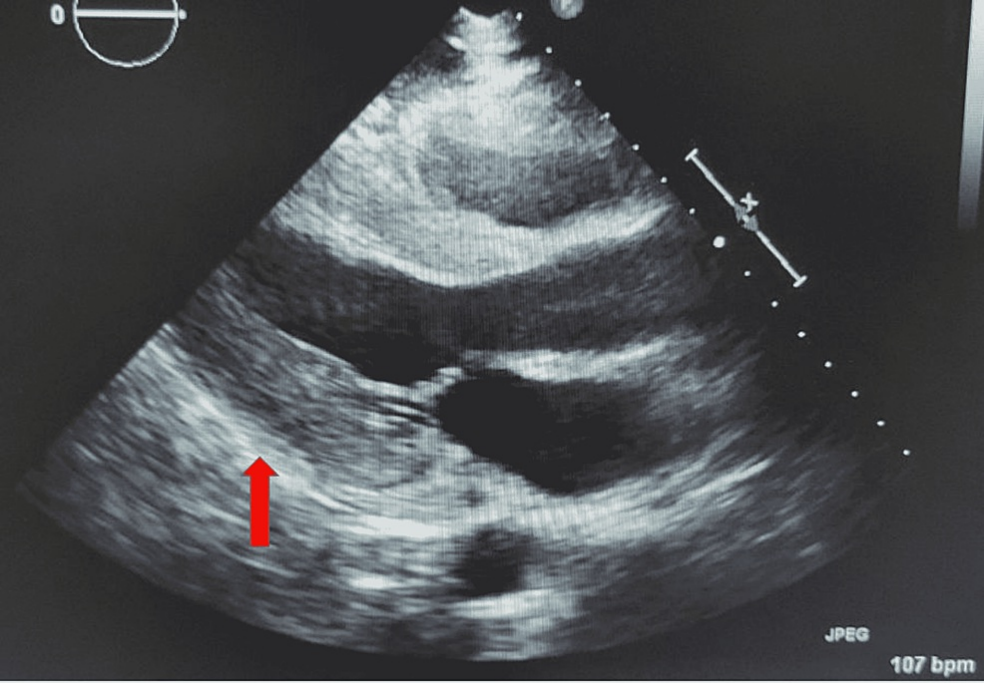
Echocardiogram post pericardiocentesis showed no pericardial effusion (red arrow).

An emergency pericardiocentesis was performed with 200 mL of serous fluid drainage with immediate hemodynamic improvement. EKG repeat showed a sinus rhythm and mild normalization of the voltage (Figure 2). The patient was admitted to the coronary care unit (CCU), and colchicine and ibuprofen were initiated for pericarditis. An echocardiogram was repeated on day 2 of the admission, showing no pericardial effusion (Figure 3). Pericardial fluid was sent for investigation to determine the cause for pericardial effusion, which showed negative bacterial and fungal cultures. In addition, serum antinuclear antibodies (ANA) were negative, so other infectious and autoimmune causes were also ruled out.

Initially, leukocytosis and erythrocytosis worsened, reaching a maximum of 32000 and 20 g/dl, respectively. Primary causes of erythrocytosis were ruled out. On day 5 of hospitalization, electrolyte abnormalities, liver enzymes, and muscle weakness worsened. Creatinine kinase (CK) level was 25000, from 145 on presentation. COVID-induced rhabdomyolysis was suspected. CK dramatically improved with IV fluids, and colchicine was discontinued. On day 7 of admission, CK level, leukocytosis, erythrocytosis, hyponatremia resolved, and liver enzymes improved. On discharge, the CK level was 6000, and all symptoms were resolved.

## Discussion

SARS-CoV-2 is the novel virus that causes COVID-19 infection. Patients commonly develop fever, upper respiratory symptoms, and pneumonia. As the disease has spread, extrapulmonary manifestations have been reported, and the COVID-19 infection showed a broad spectrum of clinical severity ranging from asymptomatic infection to multi-organ failures [5]. Cardiovascular affection can present as myocardial infarction, myocardial injury, arrhythmias, heart failure, pericarditis, thromboembolic manifestations, and myocarditis [6].

There are many causes of myopericarditis, including infectious, autoimmune, malignancy, and idiopathic causes. Out of the infectious causes, viral infection is more common. Although, in COVID-19 infection, myocarditis is an uncommon presentation, and pericardial involvement has been rarely reported, approximately 10% of cases of COVID-19 myocarditis have pericardial effusion [3]. Our case report describes the COVID-19 related-myopericarditis with pericardial effusion, cardiac tamponade, and rhabdomyolysis.

Few similar case reports have been documented. A recent case report documented a case of viral pericarditis causing a pericardial effusion, resulting in cardiac tamponade secondary to COVID-19 infection [7]. Also, Dabbagh MF et al. reported a case with large hemorrhagic pericardial effusion with echocardiographic signs of tamponade complicated by the development of Takotsubo cardiomyopathy [8].

A study by Knight DS et al. showed MRI findings of myocarditis which were reported in 13 out of 29 patients, and pericarditis in 2 out of 29 patients [9]. A similar study by Puntmann VO et al. concluded that there was cardiac involvement in 78 patients (78%), with concomitant myocardial inflammation in 60% of patients [10].

Rhabdomyolysis associated with COVID-19 has been reported in a few studies. Pathophysiology suggests muscle injury could be secondary to direct viral myositis or indirect damage secondary to an inflammatory response that causes proteolysis and fibrosis on the muscle. A study by Ali L et al. showed higher mortality in COVID-19 patients with rhabdomyolysis vs. without (47.1% vs. 26.4%) [4].

## Conclusions

Our case shows that COVID-19 infection can present with uncommon presentations like pericardial effusion and cardiac tamponade rather than respiratory symptoms, and it can be complicated with rhabdomyolysis. The management is the same as any viral myopericarditis. Further studies are warranted to better understand the pathogenesis and management of COVID-19 myopericarditis and the long-term cardiovascular consequences of COVID-19 infection.
